# Maternal Protein Restriction Altered Insulin Resistance and Inflammation-Associated Gene Expression in Adipose Tissue of Young Adult Mouse Offspring in Response to a High-Fat Diet

**DOI:** 10.3390/nu12041103

**Published:** 2020-04-16

**Authors:** Juhae Kim, Alee Choi, Young Hye Kwon

**Affiliations:** 1 Department of Food and Nutrition, Seoul National University, Seoul 08826, Korea; kjuhae05@snu.ac.kr (J.K.); ari0621c@gmail.com (A.C.); 2 Research Institute of Human Ecology, Seoul National University, Seoul 08826, Korea

**Keywords:** adipose tissue, high-fat diet, inflammation, insulin resistance, mouse offspring, protein restriction

## Abstract

Maternal protein restriction is associated with increased risk of insulin resistance and inflammation in adulthood offspring. Here, we investigated whether maternal protein restriction could alter the risk of metabolic syndrome in postweaning high-fat (HF)-diet-challenged offspring, with focus on epididymal adipose tissue gene expression profile. Female ICR mice were fed a control (C) or a low-protein (LP) diet for two weeks before mating and throughout gestation and lactation, and their male offspring were fed an HF diet for 22 weeks (C/HF and LP/HF groups). A subset of offspring of control dams was fed a low-fat control diet (C/C group). In response to postweaning HF diet, serum insulin level and the homeostasis model assessment of insulin resistance (HOMA-IR) were increased in control offspring. Maternal LP diet decreased HOMA-IR and adipose tissue inflammation, and increased serum adiponectin level in the HF-diet-challenged offspring. Accordingly, functional analysis revealed that differentially expressed genes (DEGs) enriched in cytokine production were downregulated in the LP/HF group compared to the C/HF group. We also observed the several annotated gene ontology terms associated with innate immunity and phagocytosis in down-regulated DEGs between LP/HF and C/C groups. In conclusion, maternal protein restriction alleviated insulin resistance and inflammation in young offspring mice fed a HF diet but may impair development of immune system in offspring.

## 1. Introduction.

Nutritional status during fetal and neonatal periods can lead to adverse fetal growth and render offspring vulnerable to metabolic diseases in later life [1]. Epidemiological investigations on adult offspring conceived during the Dutch famine of 1944–1945 have highlighted the association between maternal undernutrition and later disease development [2]. Although it is not clear which nutritional deficiencies are responsible for causing offspring to become nutritionally ‘thrifty’, maternal protein intake has been extensively studied based on the pivotal roles of amino acids in fetal growth and production and secretion of insulin [3]. In addition, high-quality protein intake is relatively low in developing countries [3].

A maternal low-protein animal model has been mostly used to explore the underlying mechanisms of epidemiological findings, linking the early-life nutritional environment to the susceptibility to developing the metabolic syndrome in later life [4]. In a low-protein animal model, the fetus of dams diverts nutrients to critical organs such as brain, resulting in the impaired development of the pancreas as well as other insulin-sensitive organs, including liver, muscle, and adipose tissue [5]. These affected organs alter their metabolic functions to adapt to nutritional circumstances in later life [6,7,8].

Several studies have revealed that maternal low-protein consumption aggravates glucose intolerance and systolic blood pressure in response to postnatal high-fat diet [9,10]. However, rather protective effects of maternal protein restriction against high-fat mediated inflammation [11] and glucose intolerance [12] in male offspring were also reported. Inconsistent metabolic phenotype, including body weight, adipose tissue weight, adiposity, and size and proliferation rate of adipocytes in low-protein offspring were also reported in animal models [13,14,15,16,17]. Accordingly, several human studies have reported no associations of maternal protein intake in pregnancy with offspring insulin resistance and body composition [18,19,20,21]. 

Adipose tissue regulates energy metabolism and inflammation by secreting both pro-inflammatory and anti-inflammatory adipokines, including adiponectin, leptin, tumor necrosis factor α, and monocyte chemotactic protein-1 (MCP-1) [22]. Hence, adipose tissue inflammation and insulin resistance could result in systemic insulin resistance and ectopic fat accumulation in other organs [23]. Despite the importance of adipose tissue as a key target of developmental programming, most transcriptome analyses were conducted on the liver and skeletal muscle to investigate the evidence of developmental programming by maternal low-protein consumption [24,25,26]. Little is known about the mechanisms by which maternal protein restriction regulates the morphology and function of adipose tissue. A limited number of studies have reported that altered adipocyte size [11], neuropeptide Y system [12], and beige adipocyte differentiation [15] are involved in the regulation of inflammation and insulin sensitivity in low-protein offspring in response to high-fat exposure.

In the current study, we tested the hypothesis that maternal protein restriction affects the postweaning high-fat-diet-mediated metabolic phenotype of the adipose tissue of male young adult offspring. Systemic insulin sensitivity was determined by serum glucose and insulin levels and by the homeostasis model assessment of insulin resistance (HOMA-IR). The inflammatory status was assessed by serum adipokine levels and adipose tissue macrophage infiltration. In addition, we analyzed global gene expression of epididymal adipose tissues using microarray to investigate the underlying mechanisms.

## 2. Materials and Methods

### 2.1. Animals and Diets

After five days of acclimation, six-week-old virgin female ICR mice (Orient Bio Co., Seongnam, Korea) were randomly fed either a control protein diet (20% casein) or an isocaloric low-protein diet (10% casein). Experimental diets were prepared based on the published AIN-93 formula [27] (Supplementary Materials Table S1) and were provided ad libitum for two weeks before mating and throughout pregnancy and lactation. On postnatal day three (PD3), litters were standardized to ten pups. After three weeks of lactation, male offspring of both control and low-protein fed dams were fed a high-fat diet (45% kcal from fat; #D12451, Research diets Inc., New Brunswick, NJ, USA) for 22 weeks (4 L for the C/HF group and 3 L for the LP/HF group). The subset of offspring of dams fed a control diet received a low-fat control diet (10% kcal from fat, #D12450B, Research diets Inc.) for 22 weeks as a control group (3 litters for the C/C group). Experimental scheme is shown in Supplementary Materials Figure S1. Animals were housed in a temperature- (22 ± 3 °C) and humidity- (50 ± 10%) controlled room with a 12 h dark/light cycle. The body weight of offspring was measured weekly and food intake was recorded three times a week. At the end of the experiment day, mice were fasted for 14 h and blood samples were collected by cardiac puncture under anesthesia using a combination of zoletil^®^ (Virbac, Carros, France) and xylazine (Bayer Korea, Seoul, Korea). Epididymal, perirenal, and retroperitoneal adipose tissue were collected and kept at −80 °C until use. Two offspring per each dam were selected and examined (C/C group: *n* = 6, C/HF group: *n* = 8, and LP/HF group: *n* = 6). All experimental procedures were approved by Seoul National University Institutional Animal Care and Use Committee (SNU-151019-6-2).

### 2.2. Serum Biochemical Analyses

Serum glucose and free fatty acid (FFA) levels were analyzed using a commercial colorimetric assay kit according to the manufacturer’s protocol (Asan Pharmaceutical Co., Seoul, Korea and Shinyang Diagnostics, Seoul, Korea). Serum insulin level was measured using the ELISA kit (Shibayagi Co., Shibukawa, Japan). Serum MCP-1, adiponectin, and leptin levels were also analyzed using the ELISA kits according to manufacturer’s instruction (R&D systems, Minneapolis, MN, USA). The systemic insulin resistance index was estimated by HOMA-IR with the following formula: serum glucose (mg/dL) × serum insulin (μU/mL)/405. The index of adipose tissue insulin resistance (Adipo-IR) was calculated by multiplying serum FFA level (mmol/L) by serum insulin level (μU/mL) [28].

### 2.3. Epididymal Adipose Tissue Histology Examination

Fixed epididymal adipose tissue samples were processed into 4-μm-thick paraffin sections and stained with hematoxylin and eosin (H&E) for histological evaluation. The morphology was observed under an Olympus BX50 microscope with a DP-72 digital camera (Olympus, Tokyo, Japan), and the image was captured using the Image-Pro Plus ver. 4.5 program (Media Cybernetics Inc., Rockville, MD, USA) at 200× magnification. The presence of macrophage infiltration was assessed by immunohistochemistry with an anti-F4/80 antibody (sc-52664; Santa Cruz Biotechnology, Inc., Dallas, TX, USA) using a Universal Elite ABC peroxidase kit (Vector Laboratories, Burlingame, CA, USA) according to the manufacturer’s instructions. The selective binding was visualized by DAB (diaminobenzidine-based) substrate (Vector Laboratories). The section was counterstained with hematoxylin, mounted and examined by microscopy. For quantification of crown-like structures (CLS), the cross-sectional area of CLS in each image was analyzed using the Image J software (NIH, Bethesda, MD, USA) and was expressed as % of total area.

### 2.4. Microarray Hybridization And Data Analysis

Total RNA of the epididymal adipose tissue of the representative offspring for each dam was isolated from using RNAiso Plus (Takara Bio Inc., Shiga, Japan) and RNA purity and integrity were evaluated by Agilent 2100 Bioanalyzer (Agilent Technologies, Santa Clara, CA, USA). Transcriptome profiles were analyzed using the Clariom™ S assay for mouse (Thermo Fisher Scientific, Waltham, MA, USA). cDNA was synthesized using the GeneChip^®^ Whole Transcript (WT) Amplification kit as described by the manufacturer (Thermo Fisher Scientific). The sense cDNA was then fragmented and biotin-labeled with terminated deoxynucleotidyl transferase using the GeneChip^®^ WT Terminal labeling kit (Thermo Fisher Scientific). Approximately 5.5 μg of labeled DNA target was hybridized to the GeneChip^®^ Array (Thermo Fisher Scientific) and incubated at 45 °C for 16 h. Hybridized arrays were washed and stained on the GeneChip^®^ Fluidics Station 450 (Thermo Fisher Scientific), and scanned on the GeneChip^®^ 3000 Scanner (Thermo Fisher Scientific). Data were collected using the GeneChip^®^ Command Console^®^ Software (Thermo Fisher Scientific) and were summarized and normalized with the SST-RMA (Signal Space Transformation-Robust Multichip Analysis) method implemented in Affymetrix^®^ Power Tools. Differentially expressed gene (DEG) was determined using the independent *t*-test according to the criteria of |fold change| ≥ 1.5 and *P*-value < 0.05. Hierarchical cluster analysis was performed using complete linkage and Euclidean distance as a measure of similarity using PermutMatrix (http://www.atgc-montpellier.fr/permutmatrix/). Gene-Enrichment and Functional Annotation analysis for DEGs was performed using PANTHER (http://www.pantherdb.org) and the Kyoto Encyclopedia of Genes and Genomes (KEGG, http://kegg.jp). The resulting *P*-values were adjusted using Benjamini and Hochberg’s method for controlling the false discovery rate (FDR). The pathways with an adjusted *P*-value < 0.05 were considered to be significantly enriched. R package (version 3.6.1; www.r-project.org) was used to generate a volcano plot. An association heatmap was visualized with the Morpheus platform (https://software.broadinstitute.org/morpheus).

### 2.5. Statistical Analysis

The data were expressed as mean ± SEM. For anthropometric and biochemical parameters, outliers were identified using the Grubb’s outlier test [29] and one significant outlier in serum insulin measurement was excluded. The differences between three offspring groups of each pairwise comparison (C/HF versus C/C, LP/HF versus C/HF, and LP/HF versus C/C) were evaluated by the independent *t*-test and considered statistically significant at *P* < 0.05. The association between the significantly altered anthropometric and serum parameters and expression levels of DEGs enriched in significant KEGG pathways was determined by the calculation of Pearson’s correlation coefficient. Statistical analysis was performed using SPSS software (version 22.0; IBM, Chicago, IL, USA).

## 3. Results

### 3.1. Maternal Protein Restriction Affects Body Weight and Adiposity of Adult Offspring Fed a High-Fat Diet

No differences were found in litter size (LP/HF group: 12.7 ± 1.2, the combined C/C and C/HF groups: 12.3 ± 0.1) between two groups as reported in a recent systemic review [30]. Offspring of dams fed a low-protein diet (LP/HF group) had lower body weights than offspring of dams fed a control diet (the combined C/C and C/HF groups) on PD 3 and 21 (Table 1). During postweaning HF feeding, percentage of body weight gain was higher in the LP/HF group (688.6 ± 38.6%) compared to the C/HF group (389.5 ± 17.3%), implicating a higher catch-up growth rate in offspring of protein-restricted dams. However, the final body weights were still lower compared to the C/HF group. There was no difference in food intake between C/HF and LP/HF groups (data not shown). 

Although high-fat feeding did not significantly increase body weight in offspring of dams fed a control diet (*P* = 0.057), there were significant increases in both absolute and relative weights of retroperitoneal fat mass (Table 1). Maternal low-protein feeding significantly increased the relative weight of epididymal fat mass and reduced the relative weight of perirenal fat mass. In spite of the restricted body weight gain, the relative weight of adipose tissue mass (epididymal + perirenal + retroperitoneal fat) was significantly higher in the LP/HF group compared to the C/HF group.

**Figure 1:**
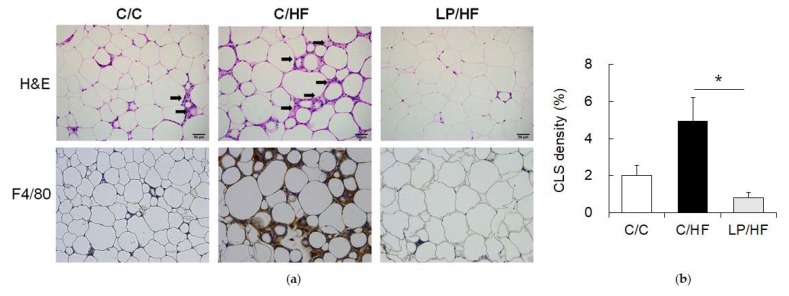
Histological analysis of epididymal adipose tissue. (**a**) Upper figures are representative H&E staining of epididymal adipose tissue section (magnification 200×). Crown-like structures (CLS) are indicated with the black arrow. Lower figures are representative immunostaining with macrophage infiltration marker F4/80 antibody (brown) from the same tissue section. Scale bar represents 50 μm. (**b**) Quantification of CLS density of the H&E stained section. Data are presented as means ± SEM (*n*= 3). * *P* < 0.05 (independent *t*-test).

We further performed histological examinations on epididymal adipose tissue. Despite the increases in the relative weight of epididymal fat mass, we observed decreased levels of aggregated macrophages in CLS around dead adipocytes in the LP/HF group compared to the C/HF group based on H&E and macrophage marker F4/80 immunostaining (Figure 1a). Quantification of H&E-stained CLS demonstrated the significant reduction of macrophage infiltration by maternal protein restriction (Figure 1b). Increased macrophage infiltration in adipose tissue is implicated in the development of obesity-related inflammation and insulin resistance [22].

### 3.2. Maternal Protein Restriction Alleviated Systemic Insulin Resistance and Adipose Tissue Inflammation of Adult Offspring Fed a High-Fat Diet

In response to postweaning high-fat diet, serum insulin level and HOMA-IR, which are used as surrogate measures of insulin resistance, were increased in offspring of dams fed a control diet (Table 2). Accordingly, serum leptin levels tended to be higher in the C/HF group compared to the C/C group (*P* = 0.058). In comparison to the C/HF group, we observed significantly lower serum insulin levels and HOMA-IR in the LP/HF group, which were comparable to that observed in the C/C group. We also observed that the Adipo-IR value, an adipose tissue insulin resistance marker, in the LP/HF group was significantly lower compared with those of C/HF group. The LP/HF group also had significantly higher adiponectin levels compared to the C/HF group. Adiponectin, one of the anti-inflammatory adipokines, is known as a positive regulator of glucose homeostasis [31]. Serum leptin and MCP-1 levels were not significantly different between the groups. 

### 3.3. Maternal Diet Altered Gene Expression of Epididymal Adipose Tissue of Adult Offspring Fed a High-Fat Diet

**Figure 2:**
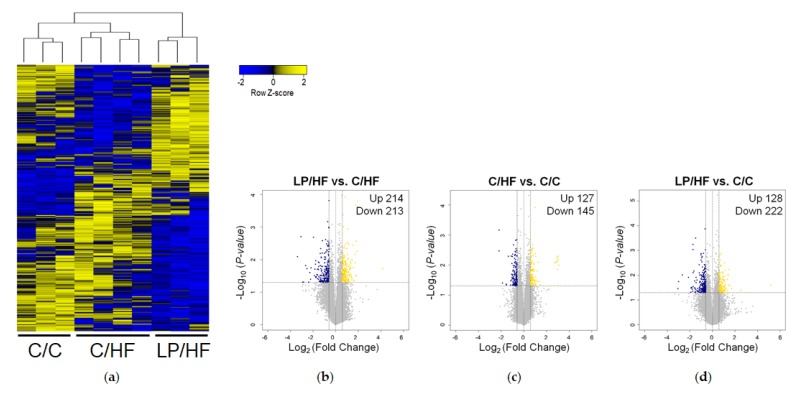
Global gene expression profile in offspring epididymal adipose tissue. (**a**) Two-dimensional hierarchical clustering of gene expression profile. Each cell represents the z-score normalized expression of differentially expressed genes (DEGs) across different samples, identified by *t*-tests between three of each pairwise comparison (*P* < 0.05, |fold change| ≥ 1.5). (**b–d**) Volcano plots (*P*-value versus fold change) of DEGs from LP/HF versus C/HF comparison (**b**), C/HF versus C/C comparison (**c**), and LP/HF versus C/C comparison (**d**). Yellow dots represent the up-regulated genes, and blue dots represent the down-regulated genes by the criteria of *P* < 0.05, |fold change| ≥ 1.5.

To figure out the mechanism involved in the increased insulin sensitivity and anti-inflammation in the epididymal adipose tissue of the LP/HF group compared to the C/HF group, we determined gene expression using microarray. Microarray analysis showed that a total of 870 genes were differentially expressed in pairwise comparison among three offspring groups (independent *t*-test, *P* < 0.05, |fold change| ≥ 1.5). Hierarchical clustering analysis of DEGs revealed that C/C and C/HF groups are separated from the LP/HF group, indicating a clear effect of maternal diet on the adipose tissue gene expression profile (Figure 2a). Maternal protein restriction significantly altered expressions of 427 genes in the epididymal adipose tissue of mice in response to postweaning high-fat diet (Figure 2b). Compared to the C/C group, there were 272 DEGs in the C/HF group (Figure 2c). In spite of similar levels of serum insulin sensitivity and anti-inflammation biomarkers, 350 genes were significantly differentially expressed between the LP/HF and C/C groups (Figure 2d).

### 3.4. Maternal Protein Restriction Regulated Gene Expression Involved in Adipokine Signaling and Inflammation of Epididymal Adipose Tissue of Adult Offspring Fed a High-Fat Diet

**Figure 3:**
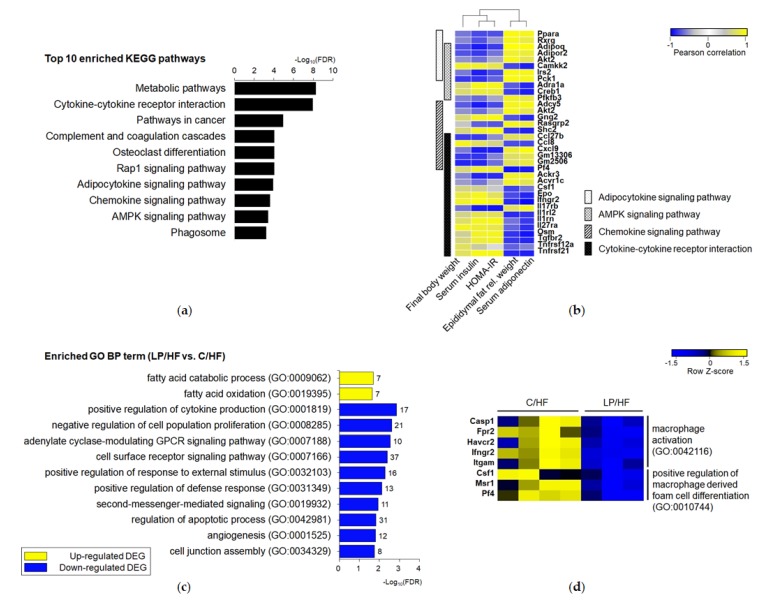
Functional enrichment analysis of the list of differentially expressed genes (DEGs) between LP/HF and C/HF groups. (**a**) The top 10 enriched Kyoto Encyclopedia of Genes and Genomes (KEGG) pathways were selected and sorted by descending order of −log10(FDR). (**b**) Heatmap showing Pearson correlation coefficients between anthropometric and serum biochemical parameters, and expressions of DEGs involved in significantly enriched KEGG pathways associated with insulin resistance and inflammation. Yellow color represents positive correlation and blue color represents negative correlation. (**c**) Gene ontology (GO) annotations of up-regulated (yellow) and down-regulated DEGs (blue). Top 10 enriched unique specific biological process (BP) terms from up-regulated DEGs and all of the unique specific BP terms from down-regulated DEGs were selected and sorted by descending order of –log10(FDR). The number of DEGs associated with the listed GO term is indicated at the edge of each bar. (**d**) Heatmap showing the z-score normalized expressions of DEGs enriched in GO terms related to macrophage. Each cell represents the expression of DEG across different samples, identified by *t*-test (*P* < 0.05, |fold change| ≥ 1.5).

To investigate the DEG-related pathways, we firstly performed KEGG pathway enrichment analysis with a total of 427 DEGs between LP/HF and C/HF groups. Results of the KEGG analysis showed 48 overrepresented pathways. Among the top 10 pathways, we observed several pathways associated with insulin resistance and inflammation, including “adipocytokine signaling pathway”, “AMP-activated protein kinase (AMPK) signaling pathway”, “chemokine signaling pathway”, and “cytokine–cytokine receptor interaction” (Figure 3a). The expression levels of DEGs that were enriched in these four annotated pathways were apparently correlated with anthropometric and biochemical parameters, including final body weight, the relative weight of epididymal fat mass, HOMA-IR, and serum levels of adiponectin and insulin (Figure 3b).

To further evaluate the altered biological functions of offspring in response to maternal low protein diet, we performed gene ontology (GO) enrichment analysis using PANTHER with consideration of the directionality of expression change. In the biological process (BP), up-regulated DEGs were enriched in total of 11 terms, including the two most “specific” terms (farthest from the root), which were “fatty acid catabolic process” and “fatty acid oxidation”. Down-regulated DEGs were enriched in a total of 133 terms including 33 specific terms. The two specific terms for up-regulated DEGs and the top 10 specific terms for down-regulated DEGs are shown in Figure 3c. “Positive regulation of cytokine production”, “positive regulation of response to external stimulus”, and “positive regulation of defense response” were highly enriched in down-regulated DEGs. Regarding terms’ associated signal transduction, “adenylate cyclase-modulating G protein-coupled receptor signaling pathway”, “cell surface receptor signaling pathway”, and “second-messenger-mediated signaling” were enriched. Besides, cell cycle regulation and extracellular environment were identified as a significant portion of the enriched terms. The list of DEGs of enriched BP terms is provided in Supplementary Materials Table S2.

Consistent with higher areas of CLS (Figure 1a,b), “macrophage activation (FDR = 0.030)” and “positive regulation of macrophage derived foam cell differentiation (FDR = 0.039)” were enriched with down-regulated DEGs (Figure 3d). Itgam (fold change = −3.01), also known as Cd11c, is one of markers for recruited adipose tissue macrophage [32]. Fpr2 (fold change = −1.59) is recently reported to be an important regulator in recruited macrophage polarization [33]. Pf4 (fold change = −2.28), a gene encoding CXC-chemokine platelet factor 4, is known as a monocyte differentiation inducer into macrophages [34].

In addition to DEGs enriched in “chemokine signaling pathway” and “cytokine–cytokine receptor interaction”, Clec4d (fold change = −10.45) and Timp1 (fold change = −8.47) were listed in the top 10 down-regulated genes between LP/HF and C/HF groups (Supplementary Materials Table S3). These two genes are known as proinflammatory genes, associated with the risk of insulin resistance [35,36]. A resistin-like alpha-encoding gene, Retnla (fold change = 3.66), is listed in the top 10 up-regulated DEGs. It was identified as an atheroprotective adipokine with a cholesterol-lowering effect [37]. Interestingly, we found five Mup genes that encoded major urinary protein, a member of the lipocalin super-family, were significantly downregulated in the LP/HF group compared to the C/HF group.

### 3.5. Adipose Tissue of Offspring Exposed to Maternal LP and Postweaning HF Diet Showed a Different Gene Profile from Those of Offspring Exposed to a Life-Long Control Diet

In spite of similar biochemical and histochemical characteristics between LP/HF and C/C groups, a total of 350 genes were extracted, including 128 up-regulated and 222 down-regulated genes as shown in Figure 2d. Therefore, we compared DEGs between two groups to get more biological insight associated with the possible dysfunction or malfunction of offspring of dams fed a restricted protein diet. When KEGG pathway enrichment of the 350 DEGs was conducted, 47 pathways were enriched with a criterion of FDR < 0.05 (Figure 3a). Among the top 10 enriched KEGG pathways, “complement and coagulation cascades”, “phagosome”, “human papillomavirus infection”, and “systemic lupus erythematosus” were highly enriched in DEGs. In addition, six pathways, including “phosphatidylinositol (PI3K)-Akt signaling pathway”, “mitogen-activated protein kinase (MAPK) signaling pathway”, “Ras signaling pathway”, and “pathways in cancer”, were related to cell proliferation and cancer development.

**Figure 4:**
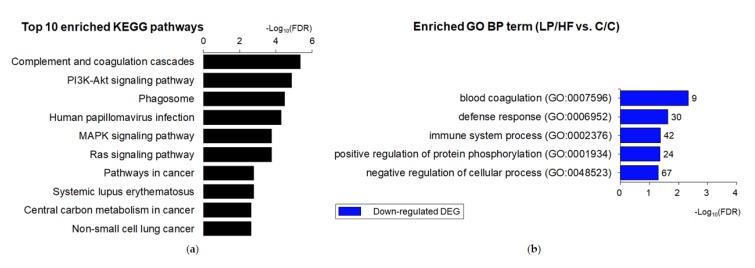
Functional enrichment analysis of the list of differentially expressed genes (DEGs) between LP/HF and C/C groups. (**a**) Top 10 enriched of Kyoto Encyclopedia of Genes and Genomes (KEGG) pathway of both up-and down-regulated DEGs were selected and sorted by descending order of –log10(FDR). (**b**) Gene ontology (GO) annotations of down-regulated DEGs based on biological process (BP). The unique specific biological process (BP) terms are selected and sorted by descending order of –log10(FDR). The number of DEGs associated with the listed GO term is indicated at the edge of each bar.

Each set of up-regulated and down-regulated DEGs was further submitted to GO enrichment analysis. In the enrichment results for biological process GO category, no GO terms were enriched in 128 up-regulated DEGs with a criterion of FDR < 0.05 (Figure 4a). Down-regulated DEGs were enriched in total of 12 terms with five specific terms, including “blood coagulation”, “defense response”, and “immune system process”. The “blood coagulation” term had three annotated parent terms: “wound healing”, “hemostasis”, and “coagulation”. Among the top 10 down-regulated DEGs, four DEGs, including Ccl8 (fold change = −7.99), Vsig4 (fold change = −3.57), Timp1 (fold change = −3.45), and Ccl12 (fold change = −3.33) were listed in enriched BP functions (Supplementary Materials Tables S4 and S5).

## 4. Discussion

Previous studies have reported inconsistent data regarding the association between adiposity and insulin resistance in the maternal low-protein model [12,38,39]. Here, we observed significantly lower inflammation and insulin resistance in the LP/HF group compared to the C/HF group in spite of increases in epididymal adipose fat mass and adiposity, which are similar to the phenotype of metabolically healthy obese (MHO) individuals [40]. Macrophage infiltration and expressions of genes associated with inflammation were apparently lower in of LP/HF group. In accordance with our observation, low-protein offspring exhibited smaller adipocytes with less macrophage infiltration compared to normal-protein offspring when fed a postnatal high-energy diet for 12 weeks [11]. In contrast to the calorie restriction model, which programs offspring to have persistent hypertrophic adipocytes [41], the low-protein model is known to program the size of adipocyte to become smaller persistently [42]. To be noted, we observed that cell cycle regulation and cancer development are enriched in DEGs between LP/HF and C/HF groups.

The “benign” increase in adiposity with increased cell proliferation and preserved insulin sensitivity was also reported in ob/ob mice with overexpressed adiponectin, in which adiponectin promotes the storage of triglycerides preferentially in adipose tissue as a peripheral “starvation” signal [43]. In the present study, serum adiponectin level was positively correlated with the relative weights of sum of fat depot (*r* = 0.740, *P* < 0.001) and epididymal fat depot (*r* = 0.835, *P* < 0.001), and negatively correlated with body weight (*r* = −0.573, *P* = 0.008) and HOMA-IR (*r* = −0.462, *P* = 0.040). Consistent with this notion, these anthropometric and biochemical changes were strongly associated with DEGs enriched in “adipocytokine signaling pathway” and “AMPK signaling pathway” in epididymal adipose tissue. Activation of the AMPK signaling pathway in adipose tissue is reported to attenuate insulin resistance [44].

Considering the degree of catch-up growth of adipose tissue, such a healthy balance between fat accumulation and inflammation observed in the present study may be temporary if mice were fed a postnatal high-energy diet for extended periods, due to the limitation of the expandability to store lipids [42]. Indeed, a longitudinal study reported that MHO, which actually has features of “benign” adipose tissue, was a transient state for older subjects with age over 40 years and less peripheral fat distribution in spite of no differences in overall adiposity [45]. Additionally, several studies reported a higher MHO prevalence in female subjects [46]. The visceral fat is reported to be tightly linked to insulin resistance leading to metabolic syndrome due to the ability to release high levels of non-esterified free fatty acids [47]. Aging-induced overall upregulation of adipokine release also promotes a chronic state of low-grade systemic inflammation [48]. 

A previous study with a long-term high-fat and high-sucrose feeding to offspring showed that nine-month-old low-protein offspring had increased relative fat mass, hyperglycemia, hypercholesterolemia, and hyperleptinemia [49]. They also reported that up-regulation of genes involved in fatty acid uptake could contribute to the increase of fat mass based on the ADIPOCHIP array designed for 89 genes related to adipose tissue differentiation and function. Although the underlying mechanism(s) are still unclear, a recent study reported the age-related loss of glucose tolerance in male low-protein offspring in response to a high-fat diet, possibly due to a decrease in brown adipose tissue (BAT) activity [16]. Compared to control offspring, low-protein offspring exhibited hyperactive BAT when 3 months and 10 months old, and less active BAT at 18 months old. Consistently, we observed that maternal protein restriction upregulated expressions of genes associated with “browning” of white adipose tissue [50,51], including Pex11a (fold change = 2.20, *P*-value = 0.009), Ucp3 (fold change = 2.75, *P*-value = 0.042), and Prdm16 (fold change = 1.71, *P*-value = 0.024) in young adult offspring. Similarly, some aspects of the metabolic phenotype progressively worsen with age in low-protein offspring fed a postweaning control diet. In a multi-time-point study, improved glucose tolerance at 9 weeks of age was diminished at 44 weeks of age in low-protein offspring [52]. In addition, low-protein offspring had an improved glucose tolerance at 3 months of age, but exhibited impaired glucose tolerance at 15-month of age [53]. Sex-related regulation of glucose metabolism was also observed in low-protein offspring. In the same study, fasting plasma insulin was significantly lower in control female offspring compared to male, but such differences were not found in low-protein offspring at 3 months of age. In 15-month-old offspring, impaired glucose tolerance was observed only in male low-protein offspring [53]. In contrast, a postweaning high-fat diet feeding study reported impaired glucose tolerance in female offspring, but improved glucose tolerance in male offspring of 21 weeks of age [12]. 

In addition, several Mup genes were found in the list of top 10 down-regulated DEGs in the LP/HF and C/HF comparison. Mup isoforms are expressed mainly in liver [54]. While the role of Mup in adipose tissue is not established well, its expression in adipose tissue has been reported to be downregulated in the diet-induced obesity model. Down-regulation of Mup was suggested as a distinct gene signature of insulin resistance, since several Mup genes were down-regulated in visceral adipose tissue of insulin resistance-prone mice before high-fat consumption as well as after high-fat consumption [55]. Mice before high-fat consumption exhibited even higher fold change differences between severe or mild insulin-resistance groups. Because we observed that several Mup genes were upregulated in the C/HF group compared to the C/C group (data not shown), further studies are required to confirm the possible transcriptomic signatures associated with maternal protein restriction. 

Apart from the comparison between LP/HF and C/HF groups, which provides information about the effects of maternal LP consumption in offspring fed a high-fat diet, any changes in gene expression observed in the LP/HF group compared to a life-long control group may deliver the metabolic alternations in response to the combination of prenatal and postnatal diet. When gene profile was compared between LP/HF offspring and C/C offspring, DEGs associated with innate immune response were altered. Furthermore, LP/HF offspring featured a significantly lowered weight of immune organ spleen compared to C/C offspring (LP/HF group: 0.088 ± 0.004 g, C/C group: 0.238 ± 0.057 g). Our findings suggest that maternal low-protein consumption followed by postweaning high-fat diet provided a detrimental effect on immune system development. Consistently, previous studies reported that thymus structure and function in adult offspring can be impaired by maternal protein restriction during pregnancy [56], and that the innate immune response can be interfered by maternal protein restriction during early lactation [57]. Accordingly, nitric oxide production of peritoneal macrophages in low-protein offspring was lower than control offspring after a postweaning high-fat diet [58]. In addition, down-regulation of complement genes was previously observed in visceral adipose tissue of the most insulin-resistant mice even before high-fat diet feeding [55]. Therefore, analysis of the underlying mechanisms by which maternal undernutrition interferes immune response in offspring would be needed.

This study would have provided more precise information about the compartmental differences in insulin resistance if it had been measured using a clamp technique, such as the “hyperinsulinemic-euglycemic clamp method”, a gold standard to validate not only systemic but also tissue-specific insulin sensitivity [59]. When comparing the changes of the systemic indicator of insulin resistance, HOMA-IR, with the changes of adipose tissue indicator of insulin resistance, Adipo-IR [28], we observed a concordant reduction of insulin resistance in high-fat-fed offspring by maternal protein consumption. Although these are serological tests, the observed results suggest a contribution of adipose tissue in the regulation of systemic insulin resistance. In the case of liver, we observed that the LP/HF group exhibited significantly lower values in the relative weights of liver and hepatic TG levels compared to the C/HF group (unpublished data), implicating the possible role of liver in alleviating systemic insulin resistance in offspring. Further studies would be needed to investigate the role of liver in the alleviation of insulin resistance in low-protein offspring in response to postweaning high-fat diet.

## 5. Conclusions

Maternal low protein consumption restricted body weight gain but increased relative adiposity in 25-week-old male offspring fed a postweaning high-fat diet. The offspring also exhibited increased insulin sensitivity and reduced inflammation, as evidenced by serum parameters as well as epididymal adipose tissue gene profile. In spite of similar metabolic phenotype, DEGs associated with innate immune response were downregulated in low-protein offspring fed a high-fat diet compared to control offspring fed a life-long time control diet, implicating the possibility of impairment of the immune system. 

The present study, which is the first analysis of the global gene expression profile of low-protein offspring adipose tissue, has provided new understanding into how maternal protein restriction regulates the morphology and function of adipose tissue in response to postweaning high-fat diet. Since the metabolic phenotypes seen at a specific time point might be transient along with the whole life of the offspring, further in-depth mechanistic studies with multi-time-point data are warranted to understand the developmental programming of adipose tissue associated with age-related dysfunction in response to postnatal overnutrition.

## Figures and Tables

**Table 1:** Effects of maternal protein restriction during pregnancy and lactation on body and adipose tissue weights of male offspring fed a high-fat diet.

	C/C	C/HF	LP/HF
Body weight (g)			
Postnatal day 3	2.62 ± 0.09		2.06 ± 0.12 ^§^
Postnatal day 20	13.20 ± 0.30		6.42 ± 0.35 ^§^
At sacrifice (25 weeks)	55.75 ± 0.96	62.12 ± 2.74	47.11 ± 0.78 *
Adipose tissue weight (g)			
Epididymal fat	1.78 ± 0.10	1.80 ± 0.27	2.48 ± 0.29
Perirenal fat	0.43 ± 0.03	0.64 ± 0.12	0.23 ± 0.02 *
Retroperitoneal fat	0.39 ± 0.02	0.73 ± 0.12 ^#^	0.50 ± 0.07
Sum of fat depots	2.60 ± 0.13	3.18 ± 0.48	3.21 ± 0.35
Relative adipose tissue weight (g/100 g body weight)			
Epididymal fat	3.20 ± 0.18	2.81 ± 0.32	5.25 ± 0.59 *
Perirenal fat	0.77 ± 0.05	1.00 ± 0.16	0.48 ± 0.04 *
Retroperitoneal fat	0.70 ± 0.04	1.14 ± 0.15 ^#^	1.05 ± 0.14
Sum of fat depots	4.67 ± 0.22	4.96 ± 0.57	6.69 ± 0.69 *

Data are presented as means ± SEM (*n* = 6−8). ^§^ Significantly different from the combined data of control offspring fed either postweaning control diet (C/C group) or high-fat diet (C/HF group) at *P* < 0.05 (independent *t*-test). ^#^ Significantly different from the C/C group at *P* < 0.05 (independent *t*-test). * Significantly different from the C/HF group at *P* < 0.05 (independent *t*-test).

**Table 2:** Effects of maternal protein restriction during pregnancy and lactation on serum biochemical parameters of male offspring fed a high-fat diet.

	C/C	C/HF	LP/HF
Glucose (mg/dL)	165.6 ± 20.7	198.4 ± 24.8	180.4 ± 20.1
Insulin (μU/mL)	13.1 ± 2.0	30.4 ± 4.4 ^#^	7.7 ± 0.6 *
HOMA-IR ^1^	5.03 ± 0.80	13.79 ± 2.40 ^#^	4.65 ± 1.34 *
Free fatty acid (mmol/L)	ND	0.88 ± 0.06	1.04 ± 0.06
Adipo-IR ^2^	ND	12.11 ± 1.86	3.83 ± 0.74 *
Adiponectin (μg/mL)	3.10 ± 0.31	2.97 ± 0.19	4.89 ± 0.28 *
Leptin (ng/mL)	24.8 ± 5.6	61.1 ± 15.7	34.6 ± 7.6
MCP-1 (pg/mL)	110.4 ± 16.0	117.9 ± 12.9	85.9 ± 10.9

Data are presented as means ± SEM (*n* = 5−8). ^#^ Significantly different from the C/C group at *P* < 0.05 (independent *t*-test). * Significantly different from the C/HF group at *P* < 0.05 (independent *t*-test). ND, not determined. ^1^HOMA-IR = serum glucose level × serum insulin level/ 405, ^2^Adipo-IR = serum FFA level × serum insulin level.

## Supplementary Materials

The following are available online at https://www.mdpi.com/2072-6643/12/4/1103/s1, Figure S1: Overview of experimental design, Table S1: Composition of experimental diet of dams, Table S2: List of DEGs of enriched GO BP terms between LP/HF and C/HF groups, Table S3: List of top 10 up-regulated and down-regulated DEGs between LP/HF and C/HF groups, Table S4: List of DEGs of enriched GO BP terms between LP/HF and C/C groups, Table S5: List of top 10 up-regulated and down-regulated DEGs between LP/HF and C/C groups.
