# Supplementary material for: Maternal Protein Restriction Altered Insulin Resistance and Inflammation-Associated Gene Expression in Adipose Tissue of Young Adult Mouse Offspring in Response to a High-Fat Diet
Source: Nutrients. 2020 Apr 16;12(4):1103. doi: 10.3390/nu12041103 (PMC7230574; doi:10.3390/nu12041103)
Supplement: Supplementary file 1 [file nutrients-12-01103-s001.pdf]

Supplementary Materials

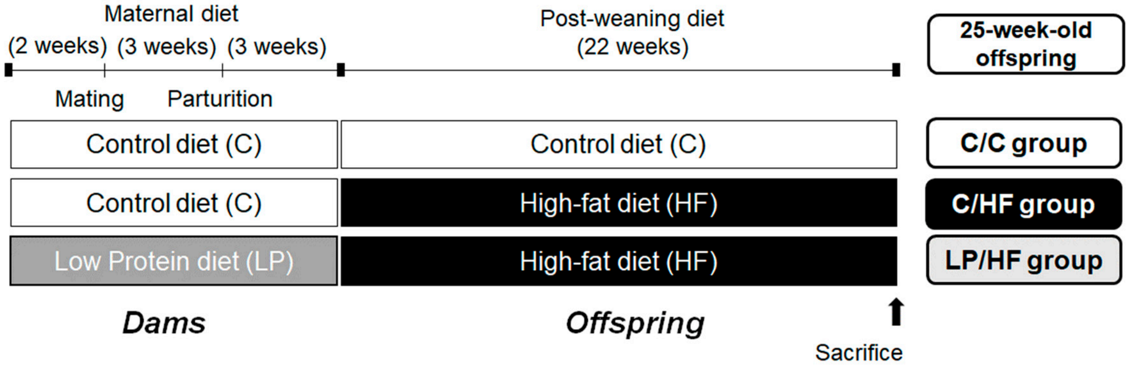

Figure S1. Overview of experimental design.

Table S1. Composition of experimental diet of dams.

Ingredient (kcal/g)	Control (C)	Low Protein (LP)
Casein [1]	200.0	100.0
L-Cystine	3.0	1.5
Corn Starch	397.5	499.0
Maltodextrin	132.0	132.0
Sucrose	100.0	100.0
Soybean Oil	70.0	70.0
Cellulose	50.0	50.0
Mineral Mix [2]	35.0	35.0
Dicalcium phosphate [3]	-	4.4
Calcium carbonate [3]	2.5	-
Vitamin Mix [4]	10.0	10.0
Choline Bitartrate	2.5	2.5
tert-Butylhydroquinone	0.014	0.014
Total	1002.5	1004.4

[1] CA160030 (Harlan, USA). [2]AIN-93G-MX (TD94046: Harlan, USA). [3]Additional amounts were added to satisfy the mouse requirement of minerals and Ca: P ratio in both groups. [4]AIN-93G-VX (TD94047: Harlan, USA).

Table S2. List of DEGs of enriched GO BP terms between LP/HF and C/HF groups.

GO biological process	FDR	DEG
**Up-regulated**		
Fatty acid catabolic process (GO:0009062)	1.97E-02	*Abcd2, Acat1, Adipoq, Gcdh, Hacl1, Pck1, Phyh*
Fatty acid oxidation (GO:0019395)	2.26E-02	*Abcd2, Acat1, Adipoq, Adipor2, Gcdh, Hacl1, Phyh*
**Down-regulated**		
Positive regulation of cytokine production (GO:0001819)	1.38E-03	*Adora2b, Casp1, Clec4n, Creb1, Cyp1b1, Epo, Fcgr3, Fzd5, Gpsm3, Havcr2, Il1rl2, Il27ra, Naip5, Osm, Pf4, Rasgrp1, Ticam2*
Negative regulation of cell population proliferation (GO:0008285)	2.41E-03	*Adora2b, Cd80, Cd9, Cyp1b1, Fam129b, Fzd5, Gas1, Havcr2, Kctd11, Lmna, Ptgir, Rassf5, Serpine2, Slfn1, Srf, Tes, Tgfbr2, Tnfrsf21, Trim35, Trpv2, Vsig4*
Adenylate cyclase-modulating G protein-coupled receptor signaling pathway (GO:0007188)	2.86E-03	*Adgrg6, Adora2b, Adra1a, Adra1a, Fpr2, Gna15, Htr1a, Npy2r, P2ry12, Pf4, Ptgir*
Cell surface receptor signaling pathway (GO:0007166)	4.07E-03	*Adgrg6, Adora2b, Angpt2, Anxa4, Atp1a3, Bcl2a1c, Bcl2a1d, Ccl8, Cd80, Clec4d, Creb1, Csf1, Egfr, Epo, Ermap, Fcgr3, Fpr2, Fzd5, Hhex, Hs1bp3, Ifngr2, Irak3, Itgam, Osm, P2ry12, Pf4, Plaur, Plek, Rassf2, Rcan2, Ror1, Sh3bp1, Shc2, Shisa6, Tgfbr2, Tnfrsf12a, Tnfrsf21*
Positive regulation of response to external stimulus (GO:0032103)	5.18E-03	*Adora2b, Alox5ap, Colec12, Csf1, Egfr, Fcgr3, Fpr2, Gpsm3, Havcr2, Irak3, Klrb1c, Lgmn, Osm, P2ry12, Rasgrp1, Ticam2*
Positive regulation of defense response (GO:0031349)	7.61E-03	*Adora2b, Alox5ap, Colec12, Egfr, Fcgr3, Fpr2, Gpsm3, Havcr2, Irak3, Klrb1c, Osm, Rasgrp1, Ticam2*
Second-messenger-mediated signaling (GO:0019932)	1.17E-02	*Adgrg6, Adora2b, Adra1a, Adra1a, Camkk2, Fpr2, Npy2r, P2ry12, Pf4, Ptgir, Rcan1, Rcan2*
Regulation of apoptotic process (GO:0042981)	1.46E-02	*Arl6ip5, Bcl2a1d, Bin1, Casp1, Cd248, Creb1, Cyp1b1, Egfr, Epo, Fam129b, Fpr2, Gas1, Hs1bp3, Il1rn, Irak3, Itgam, Lgmn, Lmna, Mical1, Naip2, Naip5, Ncf2, Osm, Pf4, Plac8, Plaur, Rassf2, Rassf5, Timp1, Tnfrsf12a, Trim35*
Angiogenesis (GO:0001525)	1.58E-02	*Angpt2, Arhgap22, Bmper, Cfh, Cyp1b1, Epo, Fzd5, Mmp19, Srf, Tgfbr2, Tnfaip2, Tnfrsf12a*
Cell junction assembly (GO:0034329)	1.73E-02	*Cd9, Cdh10, Lamc1, Nfasc, Plec, Sh3bp1, Srf, Wdr1*

Table S3. List of top 10 up-regulated and down-regulated DEGs between LP/HF and C/HF groups.

Gene symbol	Name	Fold change	*P*-value
**Up-regulated**			
S100b	S100 protein, beta polypeptide, neural	17.81	0.019
Id1	Inhibitor of DNA binding 1	3.89	0.000
Retnla	Resistin like alpha	3.66	0.005
Peg3	Paternally expressed 3	3.53	0.030
Pck1	Phosphoenolpyruvate carboxykinase 1, cytosolic	3.51	0.003
Gfra2	Glial cell line derived neurotrophic factor family receptor alpha 2	3.45	0.031
Zim1	Zinc finger, imprinted 1	3.43	0.000
Pfkfb3	6-phosphofructo-2-kinase/fructose-2,6-biphosphatase 3	3.39	0.012
Olfr1372-ps1	Olfactory receptor 1372, pseudogene 1	3.26	0.007
Nrg4	Neuregulin 4	3.10	0.045
**Down-regulated**			
Clec4d	C-type lectin domain family 4, member d	-10.45	0.008
Timp1	Tissue inhibitor of metalloproteinase 1	-8.47	0.002
Mup12	Major urinary protein 12	-7.53	0.049
Mup2	Major urinary protein 2	-7.20	0.049
Mup13	Major urinary protein 13	-6.34	0.015
Mup7	Major urinary protein 7	-5.23	0.049
Mup8	Major urinary protein 8	-4.84	0.025
Ncf2	Neutrophil cytosolic factor 2	-4.66	0.040
Msr1	Macrophage scavenger receptor 1	-4.52	0.018
Plac8	Placenta-specific 8	-3.92	0.002

DEGs are listed in descending order of |fold change|.

Table S4. List of DEGs of enriched GO BP terms between LP/HF and C/C groups.

GO biological process	FDR	DEG
**Down-regulated**		
Blood coagulation (GO:0007596)	4.54E-03	*Anxa8, Cfh, F13a1, P2ry12, Pdgfra, Pf4, Procr, Serpine2, Srf*
Defense response (GO:0006952)	2.35E-02	*Ackr2, Ahsg, Ang, C1qc, C1ra, C8a, Ccl12, Ccl8, Cfh, Clec4n, Ecm1, Egfr, Fcna, Ifit1bl2, Il1rn, Lyz2, Mrc1, Nfkb2, Pf4, Pik3cd, Pik3cg, Ppp1r14b, Rab43, Rps19, Timp1, Tnfaip8, Trim30a, Trim59, Tslp, Vamp8*
Immune system process (GO:0002376)	4.19E-02	*Ackr2, Ang, C1qc, C1ra, C8a, Ccl12, Ccl8, Cd248, Cfh, Clec1b, Clec4n, Colec12, Ctsh, Ermap, Fcna, Flt3, Fzd7, H2-M2, Hfe, Hhex, Ifi204, Ifit1bl2, Il1rn, Kcnn4, Mfap5, Mrc1, Nfkb2, Pdgfra, Pf4, Pik3cd, Pik3cg, Ppp1r14b, Rab43, Rps19, Sgpl1, Srf, Tnfrsf17, Trim30a, Trim59, Tslp, Vamp8, Zfp385a*
Positive regulation of protein phosphorylation (GO:0001934)	4.33E-02	*Ang, Ccl12, Ccl8, Dlg3, Egfr, Fam129a, Fgfr1, Flt3, Grem1, Hfe, Htr2b, Iapp, Il1rn, Inhbb, Lrrn3, Madd, Map3k13, Nmb, Osbpl8, Pdgfra, Pik3cg, S1pr2, Shc2, Tslp*
Negative regulation of cellular process (GO:0048523)	4.93E-02	*Ahsg, Ang, Anxa4, Anxa8, Arap1, Astl, C1qc, Ccl12, Creb1, Ctsh, Dlg3, Dnm1, Dok1, Dpep1, Draxin, Dusp7, Ecm1, Egfr, Fam129a, Fam129b, Fgfr1, Flt3, Fntb, Fzd7, Gas1, Grem1, Hes6, Hfe, Hhex, Hist2h2ab, Htr1b, Htr2b, Iapp, Il1rn, Inhbb, Jund, Madd, Maf, Mlx, Nbl1, Nfkb2, Nmb, Nme1, Osbpl8, P2ry12, Pdgfra, Pf4, Pik3cg, Pla1a, Plat, Rassf5, Rgs10, S1pr2, Sap30, Serpine2, Sp2, Srf, Suv39h2, Tigit, Timp1, Trim30a, Trim59, Tslp, Vamp8, Vsig4, Xrcc2, Zfp385a*

Table S5. List of top 10 up-regulated and down-regulated DEGs between LP/HF and C/C groups.

Gene symbol	Name	Fold change	*P*-value
**Up-regulated**			
Gm11096	Predicted gene 11096	36.47	0.025
Zfp600	Zinc finger protein 600	5.74	0.040
Fhit	Fragile histidine triad gene	3.26	0.035
Sfrp5	Secreted frizzled-related sequence protein 5	3.00	0.026
Rex2	Reduced expression 2	2.80	0.050
Scgn	Secretagogin, EF-hand calcium binding protein	2.78	0.006
Pck1	Phosphoenolpyruvate carboxykinase 1, cytosolic	2.71	0.024
Gm11116	Predicted gene 11116	2.65	0.010
Tmem182	Transmembrane protein 182	2.59	0.009
Pfkfb3	6-phosphofructo-2-kinase/fructose-2,6-biphosphatase 3	2.50	0.015
**Down-regulated**			
Gnal	Guanine nucleotide binding protein, alpha stimulating, olfactory type	-8.32	0.035
Ccl8	Chemokine (C-C motif) ligand 8	-7.99	0.018
Hvcn1	Hydrogen voltage-gated channel 1	-6.29	0.010
Wfdc17	WAP four-disulfide core domain 17	-4.38	0.012
Lgi2	Leucine-rich repeat LGI family, member 2	-3.74	0.048
Atp1a3	ATPase, Na+/K+ transporting, alpha 3 polypeptide	-3.72	0.033
Msr1	Macrophage scavenger receptor 1	-3.71	0.032
Vsig4	V-set and immunoglobulin domain containing 4	-3.57	0.031
Timp1	Tissue inhibitor of metalloproteinase 1	-3.45	0.037
Ccl12	Chemokine (C-C motif) ligand 12	-3.33	0.044

DEGs are listed in descending order of |fold change|.
